# Yisaipu^®^ Provide AS Patients With an Economical Therapeutic Option While Original Biologicals are More Advantageous in the COVID-19 Epidemic Situation

**DOI:** 10.3389/fphar.2021.692768

**Published:** 2021-09-06

**Authors:** Hongjuan Lu, Yuanqiong Wang, Xiuwen Wang, Xin Wu, Ling Zhou, Li Lin, Rong Sheng, Haoran Tian, Ting Li, Huji Xu

**Affiliations:** ^1^ Department of Rheumatology and Immunology, Changzheng Hospital, Second Affiliated Hospital of Naval Medical University, Shanghai, China; ^2^ School of Clinical Medicine, Tsinghua University, Beijing, China; ^3^ Peking-Tsinghua Center for Life Sciences, Tsinghua University, Beijing, China

**Keywords:** ankylosing spondylitis (AS), cost-effectiveness, COVID-19, Yisaipu®, original biologicals

## Abstract

**Objectives:** Anti-tumor necrosis factor (TNF) agents have been regarded as the most effective treatment for ankylosing spondylitis (AS) so far. However, economic factors limited the prescription of original biologicals in China. Yisaipu^®^ is a biosimilar for etanercept as pre fill syringes (PFS), which has entered China’s national medical insurance catalog for more than 10 yr and was widely used because it greatly reduced the economic burden of AS patients. Yisaipu^®^ is provided subcutaneous injection in hospital setting only. We collected clinical data of AS patients before, during and after COVID-19 epidemic, in an attempt to investigate the advantages and disadvantages of original biologicals and Yisaipu^®^ during regular follow up and COVID-19 epidemic.

**Methods:** AS patients who received original biologicals or Yisaipu^®^ in our department for more than 1 yr were included in our study. General data, demographic characteristics, disease activity, quality of life and medical compliance were collected from regular visits. The patients were followed up through telephone interviews from April 20th to 27th, 2020 about the overall impact of the COVID-19 epidemic.

**Results:** There was no significant difference in Bath Ankylosing Spondylitis Disease Activity Index (BASDAI) and Ankylosing Spondylitis Disease Activity Score-CRP (ASDAS-CRP) between the two groups. Health Assessment Questionnaire for Spondyloarthropathies (HAQ-s) showed that Yisaipu^®^ group was superior to original biological group in terms of eating, gripping and driving. In addition, the medical cost of Yisaipu^®^ was lower than that of original biologicals. The overall impact of the COVID-19 epidemic on patients of original biological group was comparatively smaller than that on Yisaipu^®^ group.

**Conclusions:** Yisaipu^®^ provided AS patients with an economical selection during regular follow-up, while original biologicals had certain advantages in the COVID-19 epidemic setting, including a longer time interval between two drug administrations and the self-injection dose form of medication.

## Introduction

Ankylosing spondylitis (AS) is a chronic autoimmune disease mainly affecting the axial skeleton with the main clinical manifestation of inflammatory lower back pain. AS can simultaneously involve the peripheral joints and extraarticular tissues, presenting as peripheral arthritis, enthesitis, ophthalmitis (mostly acute uveitis) and intestinal inflammation (Sieper and Poddubnyy, 2017). The disease is more likely to occur in young men, seriously affecting their quality of life (QOL) and working capability. In addition, AS may need a long-term course of treatment, which is a huge economical burden on individuals, families and society.

Anti-TNF therapy represents a milestone in the treatment of AS in that biologicals act fast, safely and effectively. The European League Against Rheumatism (EULAR), the Assessment of Spondylarthritis International Society (ASAS) and the American College of Rheumatology (ACR) guidelines recommend biologicals as the first line drugs for AS (Van der Heijde et al., 2017; Ward et al., 2019). Original biologicals, Infliximab (IFX) (5 mg/kg intravenous injection on week 0, week 2, week 6, once every 6 wk), Adalimumab (ADA) (40 mg once 2 wk subcutaneous injection), Golimumab (GLM) (50 mg once 4 wk subcutaneous injection)and Etanercept (ETN) (25 mg twice a week subcutaneous injection) have been approved for the treatment of AS in China. The monthly cost of full-dose administration of original biologicals averaged 3000–4000 CNY (441–588 USD), which was obviously expensive against 28,228 CNY (4151 USD) of the annual disposable income per capita (2018) in China (2019). It is generally accepted that economic factor to some extent limits the use of original biologicals in some AS patients. Yisaipu^®^ a biosimilar for ETN (25 mg twice a week subcutaneous injection), was most commonly used for the treatment of AS in China (Dongbao Zhao et al., 2021). As a tumor necrosis factor (TNF) receptor fusion protein, Yisaipu^®^ was marketed in 2005 and entered Shanghai Medical Insurance System in the same year (Scheinberg and Kay, 2012). According to the age and the state of employment of AS patients, 50–80% of the Yisaipu^®^ cost could be covered by the Medical Insurance in Shanghai, and as a result the month full-dose Yisaipu^®^ administration fee incurred by an individual patient was about 500–1000 CNY (73.5 USD∼147 USD). Compared with the original biologicals, the use of Yisaipu^®^ can greatly reduce the economic burden on AS patients so that more AS patients can afford to receive effective treatment. Although results from RCTs found out that biosimilar benefits AS patients (Xu et al., 2019), the questions whether Chinese medical insurance should cover original biologicals, or if original biologicals have more advantages than Yisaipu^®^ in real world remain unclear.

We started to establish AS patient cohort since 2016 to provide the patients regular visits, and to setup clinical database. China was the first country where COVID-19 epidemic started (Liu et al., 2020). The ongoing COVID-19 pandemic remains an important healthcare challenge for patients with chronic disease. We were very fortunate to keep in contact with most of the patients in our established through COVID-19 epidemic. We collected data of disease activity, adverse event, compliance and impact of COVID-19 epidemic on treatment of AS patients. We believed that the data of our established cohort through COVID-19 epidemic added great value to long term management of AS patients.

## Materials and Methods

Included in this study were all AS patients who received original biologicals or Yisaipu^®^ in our center and had been regularly followed up for more than a year before October 2019. All the included patients met the modified New York criteria (1984) for ankylosing spondylitis (Van der Linden et al., 1984). Before using original biologicals or Yisaipu^®^, they all had undergone regular screening tests for tuberculosis (TB), hepatitis and tumors. Treatment of AS was based on a shared decision between the patients and the rheumatologists. We collected six questions that rheumatologists and patients were concerned about when considering the use of aTNF’s (therapeutic effect, cost, safety, drug tapering and discontinuation, interval of drug administration, methods of administration). We recorded and analyzed the three most important questions choosed by rheumatologists and patients. We collected data on adverse events from two sources. One was patient’s report, and the other was laboratory tests or medical instrument exam results. After recording AE, rheumatologists evaluated the severity of AE and judged whether AE was related to biologicals. All data of cost were collected from patients report. As the proportion of Chinese medical insurance coverage varied according to the age and working status of patients, it was difficult to get accurate data of cost from physicians’ clinics. IFX and Yisaipu^®^ must be administered in our clinic, while ETN, ADA and GLM can be injected by patients themselves. Rheumatologists made appointment of next visit with patients in daily clinics, but no reminder would be sent to patients in regular practice in China. Patients’ data included their general and demographic characteristics, disease activity, BASDAI, ASDAS-CRP, Patient’s Global assessment (PGA), QOL, medical compliance, income levels and expenditures. QOL was assessed by Health Assessment Questionnaire for the Spondyloarthropathies (HAQ-S) (Zochling, 2011), including dressing, arising, eating, walking, hygiene, reaching, gripping, activities, desk job and driving, totaling 10 dimensions. Compliance was assessed by Compliance Questionnaire-Rheumatology (CQR) including 19 questions (De Klerk et al., 1999). In addition, the patients were also asked about whether they omitted or misused the drug, reasons for omitting or misusing the drug. Cost of anti-TNF treatment was also recorded according to patients’ report.

The pre-COVID-19 data were collected between Aug, 2019 to Oct, 2019, and the post-COVID-19 data were collected between Mar, 2021 to Apr, 2021 from regular face-to- face visits. The data during COVID-19 were collected by telephone interviews from April 20th to 27th, 2020. Patients were asked about the overall impact of the epidemic on them including change in the frequency of drug administration, using visual analogue scale (VAS). We defined 0 as no influence, and 10 as great influence of epidemic so that the patient could not continue therapy. The study closely followed the principles of the Declaration of Helsinki and was approved by ethics committee of the Second Military Medical University, and all patients provided written informed consent.

All obtained data were analyzed by SPSS 21.0. Continuous variables were expressed as mean ± standard deviation while categorical variables were expressed as frequency (composition ratio). Comparison between groups of normally distributed continuous variables was conducted by independent sample *t* test; comparison between two groups of continuous variables with non-normal distribution was conducted by Wilcoxon sign rank test. Chi-square test was used to compare categorical variables between the two groups. Values of *p* < 0.05 were considered statistically significant.

## Results

This study included 219 patients, of whom 41 patients received original biologicals including ENT (*n* = 4), IFX (*n* = 28), ADA (*n* = 8) and GLM (*n* = 1), and 178 patients received the Yisaipu^®.^ There was no significant difference in age, sex between patients using original biologicals and those using Yisaipu^®^, nor was there significant difference in BASDAI and ASDAS-CRP between the two groups, excepted that C-reactive protein (CRP) of Yisaipu^®^ group was lower than that of original biological group (10.6 ± 14.1 *vs* 16.4 ± 21.2). The treatment cost for anti-TNF treatment in Yisaipu^®^ group was significantly lower than that in original biological group (174.1 ± 117.4 *vs* ± 340.2 ± 279.8). The result of HAQ-S suggested that Yisaipu^®^ group was superior to original biological group in eating (0.1 ± 0.3 *vs* 0.2 ± 0.6), gripping (0.1 ± 0.5 *vs* 0.3 ± 0.6) and driving (0.9 ± 0.9 *vs* 1.4 ± 1.0) (Table 1). With respect to safety, no severe adverse event occurred throughout the treatment period in both groups. The main adverse events in original biological group and Yisaipu^®^ group were upper respiratory tract infection (14.5% *vs* 14.0%), pulmonary infection (2.4% *vs* 1.7%), herpes zoster (2.4% *vs* 0.6%), injection site reaction (2.4% *vs* 15.2%), and liver enzyme elevation (4.8% *vs* 8.4%) (Table 2).

**TABLE 1:** Patient demographic characteristics and clinical manifestations.

		Original Biologicals (*n* = 41)	Yisaipu^®^ (*n* = 178)	*p* value
Age (yr)		35.8 ± 12.4	38.0 ± 10.8	0.132
Sex (male) (*n*, %)		35 (85.4)	156 (87.6)	0.694
Marriage (*n*, %)	Unmarried	14 (34.1)	42 (23.6)	0.304
	Divorced	1 (2.4)	2 (1.1)	
	Widowed	0 (0.0)	2 (1.1)	
	Married	26 (63.4)	132 (74.2)	
Income (USD/year) (*n*, %)	<7,352.9	26 (63.4)	45 (25.3)	<0.001^a^
	7,352.9–14,705.9	13 (31.7)	59 (33.1)	
	14,705.9–29,411.8	2 (4.9)	46 (25.8)	
	>29,411.8	0 (0.0)	28 (15.7)	
Education level (*n*, %)	Primary school	2 (4.9)	4 (2.2)	0.153
	Junior school	11 (26.8)	32 (18.0)	
	High school	14 (34.1)	45 (25.3)	
	College	13 (31.7)	85 (47.8)	
	Master	1 (2.4)	12 (6.7)	
Duration (yr)		11.3 ± 8.9	10.7 ± 7.9	0.815
Follow-up (yr)		3.1 ± 2.2	3.3 ± 2.8	0.553
Cost (USD/mo)		340.2 ± 279.8	174.1 ± 117.4	<0.001^a^
CRP (mg/dl)		16.4 ± 21.2	10.6 ± 14.1	0.049^b^
BASDAI		3.3 ± 2.3	3.1 ± 1.9	0.772
ASDAS-CRP		1.9 ± 0.9	1.8 ± 0.7	0.776
PGA		3.0 ± 0.7	2.9 ± 0.8	0.808
HAQ-S	Mean	0.5 ± 0.6	0.4 ± 0.5	0.102
	Dressing	0.5 ± 0.7	0.4 ± 0.8	0.354
	Arising	0.6 ± 0.8	0.4 ± 0.7	0.087
	Eating	0.2 ± 0.6	0.1 ± 0.3	0.017^b^
	Walking	0.6 ± 0.9	0.5 ± 0.8	0.238
	Hygiene	0.4 ± 0.7	0.3 ± 0.7	0.052
	Reaching	0.8 ± 0.9	0.6 ± 0.9	0.066
	Gripping	0.3 ± 0.6	0.1 ± 0.5	0.004^b^
	Activities	0.8 ± 1.0	0.4 ± 0.7	0.099
	Driving	1.4 ± 1.0	0.9 ± 0.9	0.001^b^
	Desk job	1.0 ± 0.9	0.9 ± 0.8	0.612
Extra-articular manifestations (*n*, %)	Uveitis	5 (12.2)	30 (16.9)	<0.001^a^
	IBD	4 (9.8)	0 (0.0)	
	Psoriasis	3 (7.3)	2 (1.1)	
	None	29 (70.7)	146 (82.0)	
T-Spot (*n*, %)	Positive	0 (0.0)	26 (14.6)	0.019^b^
HBsAg (*n*, %)	Positive	0 (0.0)	10 (5.6)	0.255
Age-appropriate for work		34	157	NA
Employed (*n*, %)		18 (52.9)	143 (91.1)	<0.001^a^

a *p* < 0.001.

b *p* < 0.05.

**TABLE 2:** Adverse events of patients.

	Original Biologicals (*n* = 41)	Yisaipu^®^ (*n* = 178)
Upper respiratory infection (*n*, %)	ENT 1 (2.4)	25 (14.0)
	ADA 1 (2.4)	
	GLM 1 (2.4)	
	IFX 3 (7.3)	
Pneumonia (*n*, %)	IFX 1 (2.4)	3 (1.7)
Herpes Zoster (*n*, %)	GLM 1 (2.4)	1 (0.6)
Injection site reaction (*n*, %)	ENT 1 (2.4)	27 (15.2)
Abnormal liver function (*n*, %)	IFX 1 (2.4)	15^a^ (8.4)
	GLM 1 (2.4)	
Two or more AE (*n*, %)	IFX 1 (2.4)	7 (3.9)
	ADA 1 (2.4)	

AE, adverse effect.

a Two of which with a combination of isoniazid.

With respect to compliance, there was no significant difference in the compliance questionnaire of rheumatology (CQR) between the two groups. There were cases of omitting or misusing the drugs in the original biological group *vs* Yisaipu^®^ group (22, 53.6% *vs* 55, 30.9%). The main reasons for omitting or misusing the drugs were forgetting to use the drug (12, 29.3% *vs* 35, 19.7%), changing the frequency of drug administration spontaneously (12, 29.3% *vs* 23, 12.9%), mistaking the time of drug administration (0, 0% *vs* 4, 2.2%), and having no drugs (0, 0% *vs* 12, 6.7%). The proportion of patients who used a device to remind them of drug administration was relatively low in both groups (6, 14.6% *vs* 23, 12.9%) (Table 3).

**TABLE 3:** Compliance of patients.

	Original Biologicals (*n* = 41)	Yisaipu^®^ (*n* = 178)	*p* value
CQR	71.4 ± 10.9	70.5 ± 12.0	0.753
Omitting or misuse the drug	22 (53.6)	55 (30.9)	0.006*
Reasons for omitting or misusing the drug			
Forget to use the drug	12 (29.3)	35 (19.7)	NA
Changing drug administration frequency spontaneously	12 (29.3)	23 (12.9)	NA
Mis-remembering the time of drug administration	0 (0)	4 (2.2)	NA
No drug available	0 (0)	12 (6.7)	NA
Using a device to remind drug administration	6 (14.6)	23 (12.9)	NA

NA, not available.

**FIGURE 1:**
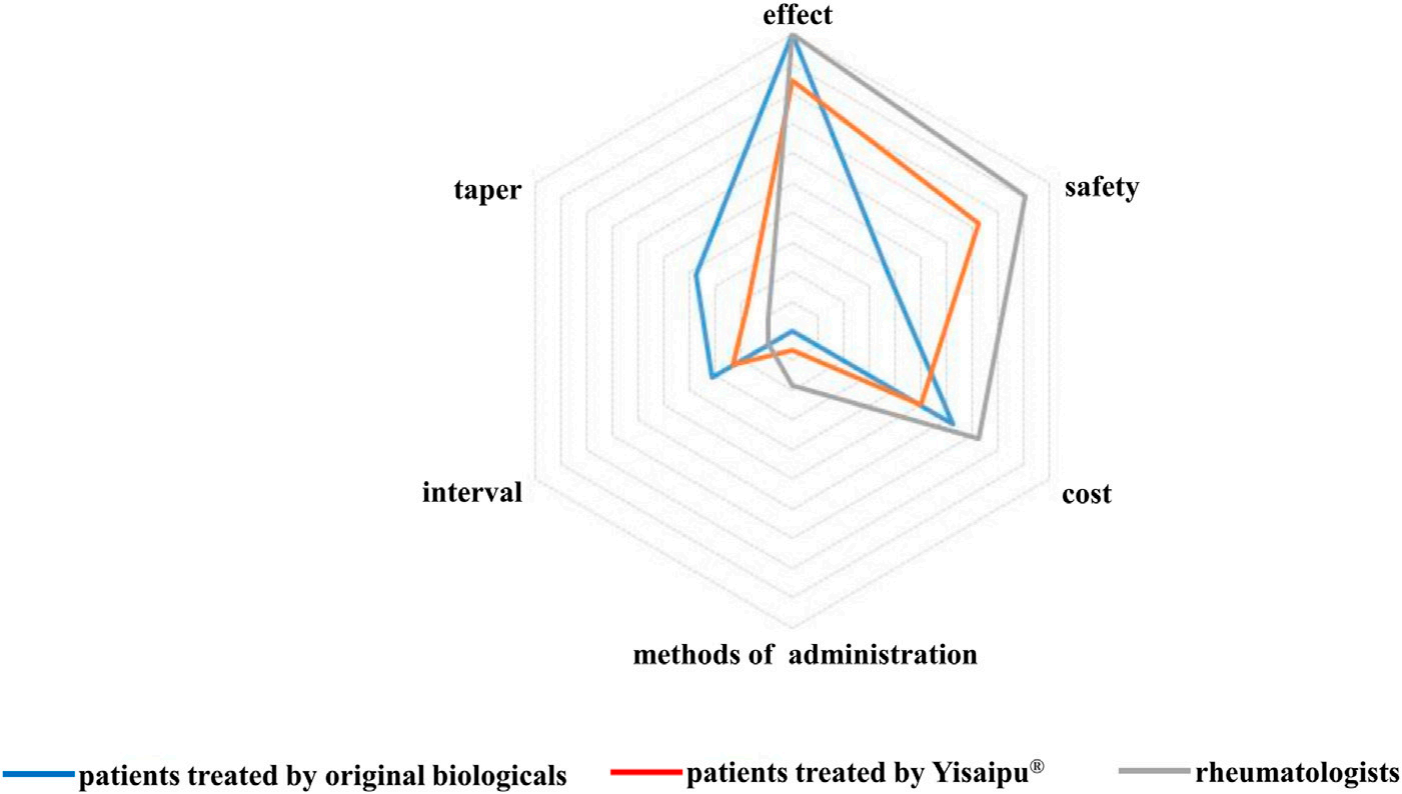
Concerned problems in original biological and Yisaipu^®^ groups, The most concerned problems in original biological group were the therapeutic effect (41, 100%), cost (25, 61.0%), safety (15, 36.6%), drug tapering and discontinuation (15, 36.6%), interval of drug administration (13, 31.7%), route of drug administration (0, 0%). The most concerned problems in Yisaipu^®^ group were the therapeutic effect (150, 84.3%), safety (129, 72.5%), cost (90, 50.6%), interval of drug administration (41, 23.0%), drug tapering and discontinuation (31, 17.4%), and route of drug administration (12, 6.7%). The most concerned issues of the rheumatologists were the therapeutic effect (11, 100%), safety (10, 90.9%), cost (8, 72.7%), way of injection (2, 18.2%), interval of drug administration (1, 9.1%), and drug tapering and discontinuation (1, 9.1%).

The most concerned problems in original biological group were the therapeutic effect (41, 100%), cost (25, 61.0%), safety (15, 36.6%), drug tapering and discontinuation (15, 36.6%), interval of drug administration (13, 31.7%), route of drug administration (0, 0%). The most concerned problems in Yisaipu^®^ group were the therapeutic effect (150, 84.3%), safety (129, 72.5%), cost (90, 50.6%), interval of drug administration (41, 23.0%), drug tapering and discontinuation (31, 17.4%), and route of drug administration (12, 6.7%). The most concerned issues of the rheumatologists were the therapeutic effect (11, 100%), safety (10, 90.9%), cost (8, 72.7%), way of injection (2, 18.2%), interval of drug administration (1, 9.1%), and drug tapering and discontinuation (1, 9.1%) (Figure 1).

From April 20th to 27th, 2020, we made telephone interviews with the 41 patients in original biological group and 150 patients in Yisaipu^®^ group. Of the 178 patients originally enrolled in Yisaipu^®^ group, 15 were unable to be contacted by telephone, five male patients and a female patient discontinued the drug for preparing pregnancy, five patients converted to original biological therapy, and two patients discontinued the drug because of upper respiratory tract infection. We evaluated the effect of COVID-19 on the treatment of AS patients from two aspects. One was subjective effect, referring to VAS of impact of epidemic on the treatment. The other was the effect on disease activity (BASDAI, PGA). We found significant difference in subjective effect (Table 4). Compared with Yisaipu^®^ group, the impact on original biological group was relatively small in terms of the visual analogue score (VAS) of patients who personally reported that the epidemic affected their treatment (2.9 ± 2.8 *vs* 5.2 ± 2.9); the proportion of patients who discontinued the use of the drug was relatively low (17.1% *vs* 33.3%), and the percentage of patients who maintained the regular frequency of drug administration was also high (80.5% *vs* 29.3%). As for the impact on disease activity, we did not find significant difference in BASDAI and PGA before, during and after COVID-19 epidemic in both groups. As majority of AS patients did not attend hospital during COVID-19 epidemic, comprehensive CQR data during COVID-19 epidemic were not available. Significant difference was fail to be found in CQR before and after COVID-19 epidemic in both groups (Table 5).

**TABLE 4:** Impact of COVID-19 on patient treatment.

	Original Biologicals (*n* = 41)	Yisaipu^®^ (*n* = 150)	*p* value
VAS	2.9 ± 2.8	5.2 ± 2.9	<0.001^a^
Withdrawal (*n*, %)	7 (17.1)	50 (33.3)	0.044^b^
No change in frequency (*n*, %)	33 (80.5)	44 (29.3)	<0.001^a^
2/3 of primary frequency (*n*, %)	0 (0)	20 (13.3)	NA
1/2 of primary frequency (*n*, %)	1 (2.4)	27 (18.0)	NA
Less than 1/2 of primary frequency (*n*, %)	0 (0)	9 (6.0)	NA

a *p* < 0.001.

b *p* < 0.05.

**TABLE 5:** Disease activity through COVID-19 epidemic.

	Original Biologicals				Yisaipu^®^			
	Period 1 (*n* = 41)	Period 2 (*n* = 41)	Period 3 (*n* = 41)	*p* value	Period 1 (*n* = 178)	Period 2 (*n* = 150)	Period 3 (*n* = 160)	*p* value
PGA	3.0 ± 0.7	3.3 ± 1.0	2.9 ± 0.7	NS	2.9 ± 0.8	3.1 ± 1.2	3.1 ± 0.9	NS
BASDAI	3.2 ± 2.3	3.2 ± 1.6	2.8 ± 1.1	NS	3.1 ± 1.9	3.1 ± 1.3	3.0 ± 1.1	NS
CQR	71.4 ± 10.9	-	71.0 ± 9.5	NS	70.5 ± 12.0	-	72.0 ± 11.1	NS

Period 1: pre-COVID-19, Aug-Oct 2019; Period 2: COVID-19, Apr 20th–27th 2020; Period 3: post- COVID-19, Mar-Apr 2021; NS, no significance.

## Discussion

The present study showed that the proportion of patients who received Yisaipu^®^ was significantly higher than that of patients who received original biologicals (81.3% *vs* 18.7%), which leaded to a major difference in number of patients between two groups. This is most probably because Yisaipu^®^ has been covered by the national and Shanghai medical insurance systems since 2005. As long-term treatment is required for AS, the treatment cost is an important factor that determines what drug AS patients prefer to use. It was found in our study that more than 50% patients in both groups were concerned about the treatment cost when they made a decision to choose original biologicals or Yisaipu^®^ (61.0%*vs*50.6%). The Task Force on the Use of Biosimilars to Treat Rheumatological Diseases (2018) pointed out that the medical insurance system, economic factors and other contextual aspects of the patients should be fully considered when original biologicals or biosimilars were chosen (Jonathan Kay et al., 2018). In the present study, the cost of Yisaipu^®^ used in our patients was covered proportionally by the insurance reimbursement sponsored by the government. While the cost of original biologicals was mainly paid by the patients personally or the families. For this reason, the medical cost of Yisaipu^®^ group was significantly lower than that of original biological group. In addition, patients in original biological group were more concerned about drug tapering and discontinuation (36.6% *vs* 17.4%) and intervals between doses (31.7% *vs* 23.0%), which may also be a reason for the higher cost of using original biologicals.

The therapeutic effect was an issue that the patients in original biological group and Yisaipu^®^ group groups were concerned about (100% *vs* 84.3%). All patients consulted whether inactive disease or moderate disease activity could be achieved by original biologicals or Yisaipu^®^ before the decision was made. It was found in our study that there was no significant difference in BASDAI, PGA and ASDAS-CRP between two groups. However, the mean CRP level in Yisaipu^®^ group was significantly lower than that in original biological group. The result of HAQ-S suggested that Yisaipu^®^ group was superior to original biological group in eating, gripping and driving. The result of working-related questionnaire showed that Yisaipu^®^ group was superior to original biological group in terms of work performance and income level. This may be due to the higher rate of drug omitting or misusing in original biological group as compared with Yisaipu^®^ group (53.6% *vs* 30.9%). These results suggested that the therapeutic effect of Yisaipu^®^ was no weaker than that of original biologicals in regular and long-term follow-up patients, and that adequate treatment with Yisaipu^®^ facilitates controlling the disease and resuming normal work on the part of the patients. The proportion of extraarticular manifestations in patients using original biologicals was higher than that of patients using Yisaipu^®^. The reason may be thatYisaipu^®^ is an TNF receptor fusion protein, while the therapeutic effect of monoclonal antibody anti-TNF agents on extraarticular manifestations such as psoriasis, uveitis and inflammatory bowel disease is better than that of TNF receptor fusion protein. With further research and development of biosimilars, monoclonal antibody biosimilars will be gradually applied to clinical use. The latest randomized double-blind controlled trial has demonstrated that the therapeutic effect, safety and immunogenicity of the biosimilar IBI301 was highly similar to those of ADA in the treatment of AS patients (Xu et al., 2019). We hope that commercial availability of these biosimilars would provide more economical therapeutic alternatives for AS patients who are complicated with extraarticular manifestations.

It was found in our study that there was a certain proportion of patients who omitted or misused the drugs in both groups. The reasons for omitting or misusing the drugs included forgetting to administer the drugs, spontaneously changing the frequency of drug administration, and mis-remembering the time of drug administration. AS is a chronic disease which need long term therapy. However, the standard chronic disease management system is still lacking. In most clinics in China, there is no nurse to remind patients for their regular visits. Some patients cancelled appointment spontaneously due to different reasons, so the frequency of drug administration was changed sometimes. The proportion of patients who used a device to remind them of the ratio of medication was relatively low in both groups. Therefore, we speculated that chronic disease management was of great importance for AS patients, and it was necessary to follow up patients regularly, provide patients education, and give them suggestions of using a notebook, calendar and alarm clock to remind them of the time of drug administration for the sake of helping them use the drugs accurately and in time. Several factors influenced the compliance of AS patients. Firstly, we did not have reminders for patients. Secondly, there were always too many patients in daily clinics, and the so that it would take patients about at least 2–3 h for each follow up. Thirdly, some patients had insurance problems after they lose their jobs. All these factors had impact on patient’s compliance. So we did not speculate that the drug had to be administered within hospital compliance was always better as compared to self administered in China.

Safety was also an issue that patients in both groups concerned about. It was found that safety was an important factor affecting the persistence of TNF inhibitor therapy (Roberto; Marchesoni et al., 2009; Caporali et al., 2018). The adverse effects of TNF inhibitors mainly included infection, increased risk of TB and hepatitis B virus (HBV) infection, injection site reaction, abnormal liver function, severe allergic reaction, autoimmune disease, new onset of psoriasis, and tumors (Fouache et al., 2009; Maxwell et al., 2015; Ramiro et al., 2017; Webers et al., 2019). China was one of the 22 countries and regions with high TB burdens, and the annual incidence of TB infection in China accounts for about 10% of the total global cases (Guo et al., 2017). The prevalence of hepatitis B in China was about 5.49%, totaling about 74.60 million people (Aparna Schweitzer et al., 2015). For this reason, all AS patients need to undergo strict screenings for hepatitis B and TB before receiving original biologicals or Yisaipu^®^. Not a single patient in the original biological group was found positive for T-spot and HBsAg. It was reported that TNF monoclonal antibodies may more strongly inhibit granuloma formation in tuberculosis as compared with etanercept (Takahiko Horiuchi et al., 2010). So doctors tend to suggest the use of TNF receptor fusion protein (Yisaipu^®^ or ETN) instead of monoclonal antibody original biologicals (ADA, IFX, GLM) in patients with positive T-spot and HBsAg for safety consideration. Abnormal liver function was found in 15 patients treated with Yisaipu^®^, two of which with a combination of isoniazid. We did not observe the newly diagnosed malignant tumor during the treatment. Data from the Swedish (Anti-Rheumatic Therapy in Sweden (ARTIS)) and Danish (DANBIO) biologics registers (ARTIS = 5448, DANBIO = 3255) indicated that treatment with aTNF was not associated with increased risks of cancer [Karin Hellgren et al., 2017]. The patients in our cohort were relatively young. We will document the data of malignancy in larger population and longer follow-up. We recorded all the adverse events, but we didn’t find severe adverse event during follow up. Most of AEs such as injection site reaction or abnormal liver function in our cohort were mild and transient, we monitored clinical and laboratory test result and continue drug administration. For infections, such as upper respiratory infection with fever or pneumonia, we stopped biological treatment, and started treatment again after infection recovery. No severe adverse event occurred in our study. The overall incidence of adverse events was relatively low, while the safety and tolerance rates were relatively high in both groups. This may be due to the strict screening before initiation of the biological or biosimilar therapy in our center. The prevalence of AE was similar to other studies [Paras Karmacharya et al., 2020]. We speculated that the prevalence of AE was relatively low both in original biologicals and Yisaipu^®^ group after regular checkups. We will keep on visit and record AE during follow up to accumulate more data on safety in real world practice.

China was the first country where Covid 19 epidemic started. The ongoing COVID-19 pandemic remains an important healthcare challenge, especially for chronic diseases. But the exact data is still scarce. Since the COVID-19 epidemic outbreaked in December 2019, the formalities for outpatient visits and hospital admission had been enhanced in accordance with the quarantine requirements, which to some extent increased the inconvenience of patients coming to the hospital. In addition, some patients feared that coming to hospital would increase the risk of being infected. Furthermore, patients’ relatives, friends or colleagues advised them not to go to hospital because of their fear to get virus. All these factors posed an impact on the original biological or Yisaipu^®^ therapy of AS patients. We were very fortunate to keep in contact with most of the patients in our established and stable cohort during and after COVID-19 epidemic. The results of our telephone interviews of the AS patients under long-term follow-up observation in our department showed that COVID19 produced some impact on the treatment of patients receiving original biologicals or Yisaipu^®^. Compared with Yisaipu® group, the impact on original biological group was relatively small. With respect to the frequency of drug administration, the proportion of patients who discontinued drug administration in original biological group was lower than that in Yisaipu^®^ group, and the proportion of patients who maintained the required frequency of drug administration was also higher. This may be due to the following two reasons. On the one hand, patients using Yisaipu^®^ and IFX had to attend hospital to get their injections. As Yisaipu^®^ was not PFS and IFX needed to be injected intravenously, both Yisaipu^®^ and IFX cannot be injected by patients themselves. Nurses in China did not provide injection treatment in patient’s home. So these patients had to attend hospital to get their injections. While original biologicals, including ETN, ADAa and GLM were PFS, which can be self administered by patients themselves, which reduces the impact of drug administration on the therapeutic outcome. On the other hand, although patients receiving Infliximab needed to go to the hospital for the prescription, the interval between two drug administrations was relatively long and therefore the impact on the therapeutic outcome was relatively small within a certain period. We did not find significant increase in BASDAI, PGA during COVID-19 epidemic. This may due to a series of measures taken by the Chinese government, which made the impact of COVID-19 on regular medical services lasted for a short period of time. It is our hope that there would be self-injection dose forms for biosimilars in the near future. Commercial availability of TNF monoclonal antibody biosimilars would bring about more convenience to AS patients. The medical insurance list in China has been under yearly adjustment according the requirement of the patients, and some original biologicals have gradually entered the lists of the national and regional medical insurance systems. In addition, the prices of original biologicals and biosimilars are on the decline. We hope that all these measures and policies would help to provide AS patients with more alternatives to choose safer and more effective, economical and convenient original biologicals or biosimilars.

This study has some limitations. First, the sample size is relatively small. We still lack standard chronic disease management system in our country, and patients loss to follow up or discontinue treatment due to economic consideration, poor compliance and ineffective therapeutic outcomes or intolerability due to adverse events. Some patients went to other hospitals for continuous treatment. We have realized the importance of chronic disease management. Our ongoing work will explore causes of patients who are unable to maintain regular long-term follow-ups, in an attempt to seek better ways for chronic disease management of AS patients. Second, the original biologicals used in this study were ENT, IFX, ADA and GLM, and tYisaipu^®^. Of them, ENT and Yisaipu^®^ are receptor fusion proteins of TNF-a, while IFX, GLM and ADA are mono-clonal antibodies of TNF-a. Although they are all TNF-a inhibitors and therefore have similar action mechanisms, there are some differences in the structure, pharmacokinetics, and frequency and method of administration between these drugs. In China, there are Geleli^®^, Anjianning^®^, Sulixin^®^ (all biosimilars for ADA) registered after 2018. Geleli^®^ has been included in Chinese medical insurance in 2021. With further research and development of biosimilars, monoclonal antibody biosimilars will be gradually applied to clinical use. We will make further comparisons between the clinical data of biosimilars and their bio-originator.

There are more than 5 million AS patients in China, and the demand for anti-TNF therapy is huge. Majority of AS patients in China chose to receive biosimilar treatment considering of economic factors. So China is one of the largest markets for biosimilar. Previous RCTs have pointed out the effectiveness and safety of biosimilar, however, the data of long - term use in real world is still scarce. The data of our cohort in regular visits and through COVID-19 will add real value to chronic disease management and to the development of biosimilar in China. Although the data from regular visits indicated that there were no significant differences between original biological and Yisaipu^®^ group in effectiveness and safety, our result showed that the overall impact of the COVID-19 epidemic on original biological group was comparatively smaller than Yisaipu^®^ group. We speculate that the development of PFS and longer injection interval biosimilar will benefit AS patients during specific circumstances such as COVID-19.

## Conclusion

In summary, the medical insurance system in China has provided AS patients with more economical therapeutic alternatives. However, original biologicals do have some advantages under the special circumstance of COVID-19, We speculate that the longer intervals and PFS may provide convenience to AS patients during COVID-19.

## Data Availability

The raw data supporting the conclusions of this article will be made available by the authors, without undue reservation.

## Abbreviations

ASDAS-CRP, Ankylosing Spondylitis Disease Activity Score-CRP; BASDAI, Bath Ankylosing Spondylitis Disease Activity Index; CRP, C reaction protein; HAQ-S, Health Assessment Questionnaire for Spondyloarthropathies; NA, not available; PGA, Patient’s Global assessment; USD, USA dollar.
